# Artificial Intelligence and Robotics in Transplant Surgery: Advancements and Future Directions

**DOI:** 10.7759/cureus.43975

**Published:** 2023-08-23

**Authors:** Syed Faqeer Hussain Bokhari

**Affiliations:** 1 Surgery, King Edward Medical University, Lahore, PAK

**Keywords:** future directions, advancements, robotics, artificial intelligence (ai), transplant surgery

## Abstract

This editorial explores the transformative impact of artificial intelligence (AI) and robotics in transplant surgery. By merging robotic precision with AI analysis, this integration enhances organ transplantation outcomes. AI algorithms scrutinize patient data, elevating organ compatibility during allocation. Robotic systems such as the da Vinci Surgical System enable intricate operations with reduced complications and faster recovery. AI-driven post-transplant monitoring identifies early rejection signs, while tailored immunosuppressive regimens enhance patient care. Future prospects encompass predictive organ availability, telemedicine-enabled expertise dissemination, bioengineered organs, and personalized immunosuppression. Ethical considerations include privacy and algorithmic bias. In striking a balance, responsible AI and robotics application can revolutionize transplant surgery, offering a brighter future for patients in need.

## Editorial

Introduction

The field of medicine has experienced a revolutionary transformation through the advent of transplant surgery, bringing newfound hope to patients struggling with end-stage organ failure. Robotics and artificial intelligence (AI) developments have significantly improved the outcomes and success rates of transplant surgeries. Transplant surgeons have overcome several obstacles by integrating the accuracy and precision provided by robotic equipment with the analytical skills of AI algorithms, opening the door for an era in which organ transplantation is more effective and accessible [1]. This editorial examines the advancements achieved recently in AI and robotics in the field of transplant surgery, highlighting their dramatic effects on patient care and examining the potential future possibilities in this quickly developing field.

Advancements in AI and robotics

AI and robots have significantly improved transplant surgery, providing excellent outcomes in a variety of transplant-related processes. First, AI algorithms can examine a large quantity of patient data, including medical history, genetic variables, and donor features, to maximize organ matching in the selection and allocation of organs. As a result, informed judgments are made by transplant surgeons, increasing the likelihood of a successful transplant and lowering rejection rates [2].

Moreover, robotic-assisted surgery has transformed the way transplant procedures are performed. With the improved dexterity, stability, and precision that robots offer, surgeons can carry out intricate tasks with more precision. Robotic technologies, such as the da Vinci Surgical System, have been effectively used in kidney and liver transplant procedures, decreasing blood loss, lowering surgical stress, and speeding up patient recovery.

Furthermore, AI-driven technologies have also shown promise in post-transplant care. AI systems may identify the earliest indications of transplant rejection or complications by continuously monitoring vital signs, laboratory results, and patient-reported data. Early identification allows for prompt care, which improves patient outcomes and may even prolong the life of donated organs. Additionally, personalized immunosuppressive treatment regimens can be made possible by AI-powered prediction models, offering optimal dosing and minimal adverse effects [3].

Future directions

While the advancements in AI and robotics in transplant surgery are impressive, there are still exciting avenues to explore for future development. Employing machine learning methods to forecast organ availability is one such approach. AI systems can calculate the possibility of organ availability in various areas by examining historical data on organ donation trends. This information can aid in better planning and resource allocation for transplant centers, potentially reducing waiting times and increasing the chances of timely transplantation.

Additionally, there is a lot of promise for extending access to transplant expertise through the combination of AI and robots with telemedicine [4]. Transplant surgeons can support treatments in remote areas where access to specialist surgical care may be restricted by employing remote consultation and guidance provided by robotic equipment. This strategy can reduce the requirement for patient travel while enhancing results between transplant facilities and patients.

Another exciting area of research is the development of bioengineered organs and tissues. In order to improve the structural and functional qualities of bioengineered organs, AI algorithms can help in the design and production of biomimetic scaffolds. The exact construction and transplanting of these designed tissues can subsequently be performed by robotic devices, possibly resolving the issue of a lack of organ donors and cutting the length of the transplant waiting list.

AI-driven decision support systems can also help transplant surgeons control immunosuppression in a tailored manner. AI algorithms can provide customized immunosuppressive regimens that are catered to the exact requirements of each patient by combining data from numerous sources, including genetic profiles, medication metabolism information, and real-time patient monitoring. This strategy may lessen issues, lower the chance of rejection, and improve long-term graft survival [5].

The use of AI and robotics in transplant surgery must, however, be balanced with ethical concerns. Careful consideration must be given to issues including data privacy, algorithm bias, and the possibility of excessive dependence on automation. It is imperative to strike a balance between the advantages of new technologies, and patient safety, justice, and the preservation of rationality and compassion.

The field of transplant surgery can greatly benefit from the development of AI and robotics. These technologies have shown tremendous promise in improving patient outcomes and boosting the effectiveness of transplantation across the entire spectrum, from donor allocation and selection to surgical techniques and post-transplant care. Future directions in the field's development include anticipating organ availability, expanding availability through telemedicine, developing bioengineered organs, and handling immunosuppression on an individualized basis. However, careful consideration of ethical implications is essential. By embracing these advancements responsibly, we can harness the power of AI and robotics to revolutionize transplant surgery and ultimately improve the lives of countless patients awaiting life-saving organ transplants.
